# Hand-book of Practical Medicine

**Published:** 1887-04

**Authors:** 


HANDBOOK OF PRACTICAL MEDICINE. *By* DR. HERMAN EICHHORST, *Professor of Special Pathology and Therapeutics and Director of the University Medical Clinic in Zurich. Vol. iii.: Diseases of the Nerves, Muscles and Skin, with 157 wood engravings; pp. 390. New York:* WILLIAM WOOD & COMPANY. 1886. CHICAGO. W. T. KEENER.

The third volume of Professor Eichhorst's most excellent work upon the Practice of Medicine, is issued by William Wood & Company, as the sixth volume of " Wood's Library " for 1886.

In section seven, "Diseases of the Nervous System" are more fully treated of in this than in any other text-book on practice. The first part is devoted to diseases of the peripheral nerves, part second to diseases of the spinal cord, part third to diseases of the medulla oblongata, part fourth to diseases of the brain, part fifth to diseases of the sympathetic. Diseases of the muscles are crowded into a space of only six pages. Section seven, comprising 62 pages, is alloted to " Diseases of the Skin."

Under the head of Diseases of the Peripheral Nerves, the pathology is very clearly and correctly given; the symptomatology is clearly and concisely stated, and the electrical degenerative reactions so fully explained that they will be easily understood by one who has been puzzled by the descriptions in Erb's and other books. In this section the treatment is chiefly electrical; medical treatment is not fully enough explained.

Diseases of the spinal cord, brain and meninges are clearly described. Among special diseases we desire to call attention to the excellent manner in which tabes dorsalis, spastic spinal paralysis, progressive spinal muscular atrophy, amyotrophic lateral sclerosis, cerebral hæmorrhage, tumors within the cranial cavity and the diagnostic preliminary remarks on diseases of the brain are given.

Bend's hypertrophy of muscles is too briefly described, and its treatment receives but three lines.

Diseases of the skin are given in a condensed form, with the nomenclature of Kaposi and with that author's treatment. The section is comprehensive enough to meet the requirements of the general practitioner.

The translation is free, giving a smooth English text. The original work has suffered somewhat by the omission of what the translator, perhaps, thought unimportant paragraphs. A serious fault occurs in the repeated omission of the names of authors whose names appear in the original text.

The printing has been carefully done upon good paper, making a handsome volume. The wood cuts are valuable aids to the interpretation of the text, but are not so distinct and clear as in the original. F. B.

